# A *Atractylodes lancea* polysaccharide inhibits metastasis of human osteosarcoma U‐2 OS cells by blocking sialyl Lewis X (sLe^x^)/E‐selectin binding

**DOI:** 10.1111/jcmm.15870

**Published:** 2020-09-28

**Authors:** Kaihua Zhong, Shuxin Fan, Shujun Yao, Haibin Xu, Suping Bai

**Affiliations:** ^1^ Department of Orthopedics Zhoukou City Central Hospital Zhoukou China; ^2^ Department of Orthopedics The First Affiliated Hospital of Xinxiang Medical University Weihui China; ^3^ School of Pharmacy Xinxiang Medical University Xinxiang China

**Keywords:** apoptosis, *Atractylodes lancea*, metastasis, polysaccharide, sialyl Lewis X (sLe^x^)/E‐selectin, U‐2 OS human osteosarcoma cells

## Abstract

In this study, a new water and alkaline‐soluble polysaccharide (ALP), with an average molecular weight of 6.63 × 10^4^ Da, was successfully purified from the rhizomes of *Atractylodes lancea*. GC analysis demonstrated that ALP was a kind of glucan. The effect of the ALP on the interaction between E‐selectin and sialyl Lewis X (sLe^x^) was examined in human osteosarcoma U‐2 OS cells. It was obvious that the expression of sLe^x^ antigen on the surface of U‐2 OS cells was visible under fluorescence microscopy. The addition of ALP (0.5, 1 and 2 mg/mL) resulted in a marked inhibition on the adhesion, migration and invasion of U‐2 OS cells to human umbilical vein endothelial cells (HUVECs), which was achieved by the decreased sLe^x^ expression on U‐2 OS cells. Additionally, the induction of apoptosis can be observed in U‐2 OS cells following ALP treatment using TUNEL staining and Annexin V‐FITC/PI double‐staining analysis on flow cytometry. In conclusion, these results indicated that ALP exerted anti‐metastatic activity towards osteosarcoma cells via inhibition of sLe^x^/E‐selectin binding, which suggested that ALP could be a potent agent for human osteosarcoma intervention.

## 1 INTRODUCTION

Osteosarcoma (OS), as the most common aggressive bone tumour, frequently threatens children, adolescents and young adults with <20% survival rate for metastatic OS patients worldwide.^1^, ^2^ Despite of some advances in the diagnosis and treatment of OS, the high local relapse and distant bone invasion, or distant pulmonary metastases contributes to high mortality and poor prognosis.^1^ Badly, sometimes the pain of OS was often mistaken as common conditions such as ‘growing pains’.^3^ Therefore, a comprehensive understanding of the mechanisms that leads to the development of OS chemoresistance and metastatic recurrence is fundamental to develop novel therapeutic agents with less severe side effects for OS patients.

Metastasis is the major cause of mortality in patients with a variety of cancers and occurs as a complicated process involving cancer cell adhesion, invasion and migration.^4^ During this process, cell adhesion is one of the most important events.^5^ To date, it has been widely accepted that sialyl Lewis X (sLe^x^)/E‐selectin‐mediated tumour metastasis are identified in many types of carcinoma including colon cancer,^6^ lung carcinoma^7^ and melanoma.^8^ Accordingly, any E‐selectin antagonist blockading the adhesion of sLe^x^ and E‐selectin can decrease tumour cell motility and metastasis. A number of evidences have shown the synthesized anti‐adhesion peptides are limited because of their short half‐life and high dosage required.^9^ To eliminate or ameliorate this drawback, a new series of derivative of synthesized peptides was designed and synthesized peptides with more repeat sequence exhibit a stronger anti‐metastasis effect than non‐repeat peptides.^10^, ^11^ This clue throw light on us to search if there is some natural polymers or its chemical derivative owning the same properties as repeated peptides, but with long half‐life and long‐lasting stability.

One of the most successful natural polymers is polysaccharide and it is easily accessible from natural source. The rhizomes of *Atractylodes lancea* (Thunb.) DC. are a very popular traditional Chinese medicine and have been used extensively to treat several disease including digestive disorders, influenza night blindness and rheumatic diseases.^12^, ^13^ Previous pharmacological studies have indicated that essential oils and aqueous extract from *A lancea* had promising apoptosis‐inducing activity towards human cancer cells.^14^, ^15^ As far as we are aware, polysaccharides are principal components dissolved in aqueous extract. Increasing evidence has demonstrated that *A lancea* polysaccharides possessed intestinal immune system modulating activity ^16^, ^17^; however, until now, the study regarding antitumour and anti‐metastatic activities of *A lancea* polysaccharide are not fully investigated, let alone for OS. In an effort for discovering novel drug which were responsible for the antitumour activities of *A lancea*, we intend to purify one water‐soluble polysaccharide from this plant and examine its antitumour and anti‐metastatic effects on human osteosarcoma cell line U‐2 OS. Furthermore, the effect of this polysaccharide on the adhesion of sLe^x^ on U‐2 OS cells to E‐selectin on activated human umbilical vein endothelial cells (HUVECs) was also evaluated in U‐2 OS cells.

## 2 MATERIALS AND METHODS

### 2.1 Materials and chemicals

*Agrimonia pilosa* was purchased from the local Drug store in Zhoukou city of China. Trifluoroacetic acid (TFA), dimethyl sulphoxide (DMSO), monosaccharide standards with >98% purity (glucose (Glc), galactose (Gal), rhamnose(Rha), arabinose (Ara), xylose (Xyl), fucose (Fuc), ribnose (Rib), mannose (Man), glucuronic acid (GlcA), and galacturonic acid(GalA)) and T‐series Dextran standards (2000, 500, 70, 10, 5 kD) were purchased from Sigma‐Aldrich (St. Louis, MO). Dialysis tubes with a molecular weight cutting off (MWCO) of 3500 Da were from Hangzhou Sijiqing Company (Hangzhou, China). DEAE Sepharose Fast Flow and Sephacryl S‐300 were obtained from GE Healthcare Bio‐sciencesCo. (Piscataway, NJ). Dulbecco's Modified Eagle's medium (DMEM) and foetal bovine serum (FBS) were purchased from Gibco/Invitrogen (Grand Island, NY). All other chemical reagents used in this experiment were of analytical grade.

### 2.2 Extraction and purification of polysaccharide from rhizomes of *A lancea*

The dried rhizomes of *A lancea* (1000 g) were first ground into powder in a disintegrator and then refluxed with 95% ethanol to get rid of lipophilic constituents, such as pigments, monosaccharides, oligosaccharides and other small molecule impurities. Subsequently, the refluxed residues were filtrated and washed until without phenol‐sulphuric acid reaction and then were immersed in 10 L of 2% NaOH aqueous solution overnight at room temperature for three times. After the extract solution was combined and filtered through line cloth, the resulting suspension was added with hydrochloric acid (0.1 M) to neutralize the redundancy of NaOH. The supernatant was concentrated to 1/8 of the original volume with a rotary evaporator under reduced pressure and subjected to twice Sevag reagent (chloroform: n‐butyl alcohol = 4:1, v/v) extraction to remove protein. The yielding water layer was collected, concentrated and precipitated with three volumes of anhydrous ethanol at 4°C overnight, followed by vacuum drying to give crude *A lancea* water and alkaline‐soluble polysaccharides (CALAP, 52.34 g).

The CALP (5 g) was dissolved in deionized water (10 mL) and filtered through 0.22 μm membrane. The supernatant was loaded to a DEAE Sepharose Fast Flow column (2.0 × 50 cm) and eluted with distilled water followed by 0.25, 0.5 and 1 M NaCl aqueous solutions respectively at a flow rate of 2.0 mL/min. Each fraction (10 mL/tube) was collected by monitoring the carbohydrates content by phenol‐sulphuric acid method at 490 nm.^18^ Only one fraction (CALP) was obtained from the distilled water eluate. Then, this water‐eluted fraction was further purified by size‐exclusion chromatography on a Sephacryl S‐300 gel column (1.6 × 100 cm) eluting with 0.15 M NaCl at 1 mL/min, yielding a purified polysaccharide (ALP, 116.5 mg).

### 2.3 Molecular weight and chemical compositions analysis of polysaccharide from *A lancea*

The total carbohydrate content of polysaccharide was determined using the phenol‐sulphuric method using glucose as the standard.^18^ The protein content of polysaccharide was measured by Bradford assay using BSA as the standard.^19^ The uronic acid content of the polysaccharide was analysed by the m‐hydroxydiphenyl method with d‐glucuronic acid as standard.^20^

The homogeneity and molecular weight of the polysaccharide were determined by high performance gel permeation chromatography (HPGPC) on a Waters 1525 HPLC system (Waters) equipped with a Waters 2414 refractive index detector (RID) and a TSK‐GEL G4000PWxlcolumn (7.8 × 300 mm × 5 μm). The operation conditions were set as follows: injection volume: 10 μL; mobile phase: 0.1 mol/L NaNO_3_; flow rate: 0.9 mL/min; column temperature: 35°C; detecting temperature: 45°C. The elution time (T) of T‐series Dextran standards (2000, 500, 70, 10, 5 kD) was plotted against their respective molecular weights to generate a regression equation under the conditions described above.

The monosaccharide composition of the polysaccharide was determined by GC method as recorded by Qin et al,^16^ with some minor revision. Briefly, 5 mg of dried samples was hydrolysed with 2 mL of 2 M TFA at 120°C for 2 hours to release monosaccharide. After the removal of the excess acid, the hydrolysates were then acetylated with 1 mL of pyridine and 1 mL of acetic anhydride in a water bath at 90°C for 20 minutes. The affording alditol acetate was further analysed on an Agilent7890A system gas chromatography fitted with a DB‐5 MS capillary column capillary column (30 m × 0.25 mm × 0.25 μm) and a flame ionization detector (FID). The reference of standard monosaccharide (Glc, Gal, Rib, Rha, Fuc, Man, Ara and Xyl) was derivatized under the same conditions and monosaccharide composition of sample was identified by matching with the retention times of the monosaccharide standards with inositol as the internal standard. The column temperature was initially programmed at 100–220°C at a rate of 5°C/min, increased to 240°C at a rate of 2°C/min, and then held at 240°C for 2 minutes, followed by gradient increment to 280°C at 10°C/min. The N_2_ was used as the carrier gas at a constant flow rate of 20 mL/min.

### 2.4 Detection of sLex by fluorescence microscopy

The presence of sLe^x^ on the surface of cancer cells was determined using monoclonal antibodies specific for its epitope under fluorescence micrcscope examination according to the previous method.^7^ At first, the 6‐well glass slide was added with cancer cells (100 μL, 2 × 10^5^) and treated with 2 mM EDTA at room temperature for 30 minutes. After removing the floated cells, the adhered cells in each slide were fixed with 5% formalin‐PBS solutions for 5 minutes, and then added with 50 μL of PBS for another 15 minutes incubation. After washing with PBS twice, 50 μL of monoclonal antibodies CSLEX1 (anti‐sLe^x^, 1:500), KM93 (anti‐sLe^x^, 1:100), KM231 (anti‐sLe^a^, 1:100) solutions or PBS were added and incubated overnight at 4°C. Next day, the slide was washed with PBS for three times and the supernatant was discarded. The slides containing precipitated cells were cultured with 50 μL of FITC‐conjugated goat antimouse IgM/IgG antibody (1:200) at the room temperature for 2 hours, followed by washing with PBS twice. Finally, attached cells on the slides were imaged under a fluorescence microscope (Eclipse TS100, Nikon, Tokyo, Japan).

### 2.5 Cell adhesion experiments under static conditions

Adhesion assay was performed as described by Yue et al,^21^ with slight modification. Briefly, HUVECS (100 μL, 2 × 10^5^ cells/well) in exponential phase of growth was incubated in 96‐well plates with 5% CO_2_ at 37°C. When the cell monolayer was formed, 5 ng/mL TNF‐α was added to stimulate the E‐selectin production. After 4 hours, ALP solution in the concentration of 50 μg/mL was added and co‐cultured with HUVECs monolayer for anther 48 hours. For the ligand antagonist assay, polysaccharide sample was replaced by a neutralizing antibody for E‐selectin and treated under same conditions. After two washes with phosphate‐buffered saline (PBS), cancer cells (200 μL, 5 × 10^4^ cells/well) stained with 5 μM of Calcein AM were added to HUVECs and incubated at room temperature with rotation (120 rpm) for 4 hours. The reaction was terminated by the addition of 95% ethanol/PBS (200 μL, 1:1) in each well at 37°C for 30 minutes. Thereafter, HUVECs were gently rinsed DMEM medium containing 10% foetal bovine serum twice to remove the non‐adhesion cells. The bottoms of 96‐well plates were photographed by a microscope in five fields at a magnification of ×200. The absorbance value was then measured at 520 nm on a microplate reader. The HUVECs was treated by vehicle was used as the control. 200 μL of Calcein AM labelled cell suspension was set as 100% and the percentage of adhesion was calculated by reference to it. The rate of adhesion was calculated as below: adhesion rate (%) ＝ the mean OD value of the samples/ the mean OD value of the control × 100%.

### 2.6 Cell invasion and migration assays

The cell migration and invasion assay was performed as described previously^22^ with some modification. For invasion assay, the upper portion of Transwell chambers was coated with the HUVECs (5 × 10^4^ cell/well) for 1‐3 days to from a continuous cell monolayer. Before adding sample, 20 μL TNF‐α (5 ng/mL) was added into each cell at 37°C for 4 hours. Then, different sample solution was suspended in the 24‐well plate and incubated for 48 hours. After two washes with PBS, cancer cells were added to the upper chamber at a density of 5 × 10^4^ cells/well in 200 μL of serum‐free medium and the lower chamber was filled with medium containing 10% FBS as chemoattractant. After incubation at 37°C for 4 hours under in a humidified atmosphere containing 5% CO_2_, the non‐invading cells were gently removed from the upper chamber and the cells that penetrated to the lower side of HUVECs membrane were rinsed with PBS and stained with 0.2% Crystal violet in 2% ethanol (15 minutes). These stained cells were counted under a fluorescence microscope in five randomly selected fields. The rate of migration and invasion was expressed as the below formula: the percentage of migration rate (%) = [(migration cells treated/migration cells control) × 100]. For migration assay, all experimental operations were conducted in the same condition except that no HUVECs coating on polycarbonate filters.

### 2.7 Detection of sLex by flow cytometry analysis

To qualify the expression change of sLe^x^ on the surface of U‐2 OS cells by ALP, flow cytometry analysis was performed as previously described.^23^ Briefly, U‐2 OS cells were allowed to grow until logarithmic phase and adhering to bottom of 6‐well culture plates and then added with ALP (0.5, 1 and 2 mg/mL) for 48 hours. Afterwards, the cells (1 × 10^6^) were enriched, washed and cultured with mAbs to sialyl Lewis antigens CD15s or PBS (control) for another 1 hour followed by the exposure to FITC‐conjugated goat antimouse IgM/IgG antibody for 30 minutes at 4°C. Eventually, these cells were subjected to analysis on a FACSCalibur flow cytometer. Data were expressed as the mean fluorescence intensity or the number of positive cells. Each experiment was performed in triplicate.

### 2.8 TUNEL/DAPI staining

Apoptotic cell death was examined by the TUNEL method using a commercially available In Situ Cell Death Detection kit following the manufacturer's protocols. After removal of cell culture media, the cells from different group were washed twice with PBS and then fixed with 4% paraformaldehyde for 30 minutes at room temperature. Following 3 × PBS wash, cells were permeabilized with 0.1% Triton X‑100 in 0.1% sodium citrate on ice for 2 minutes. Subsequently, the cells were stained with TUNEL reaction buffer for 1.5 hours in the dark at 37°C and then subjected to the incubation with DAPI (1 μg/mL) at 37°C for 5 minutes to stain the cellular nuclei. Positive cells were visualized, counted and analysed under an Olympus fluorescence microscope (Olympus Corp., Tokyo, Japan) at 200× magnification. The percentage of apoptotic cells were calculated by dividing the number of TUNEL‑positive cells (dead cells) by DAPI‐stained cells (viable cells) × 100%. Three independent experiments were then averaged and statistically analysed.

### 2.9 Annexin V‐FITC/PI double‐staining analysis

To qualify the apoptosis rate, apoptotic cells stained with Annexin V‐FITC/PI were subjected to flow cytometry analysis according the manufacture's protocols.^24^ Briefly, following different ALP treatment, cancer cells were collected, rinsed twice with PBS and suspended in binding buffer. Approximately 5 μL Annexin V‐FITC and 5 μL PI were then added to the cell suspension and kept in the dark for 15 minutes at the room temperature. After twice washing with PBS, at least 1 × 10^4^ cells were loaded on a FACSCalibur flow cytometer for data collection and three separate replicates were done for each clone.

### 2.10 Statistical analysis

The data are presented as means ± SD The differences between the groups were analysed with analysis of variance (ANOVA). *P*‐values of <0.05 were accepted to be statistically significant.

## 3 RESULTS

### 3.1 Isolation, purification and characteristic of different polysaccharide from *A lancea*

**FIGURE 1:**
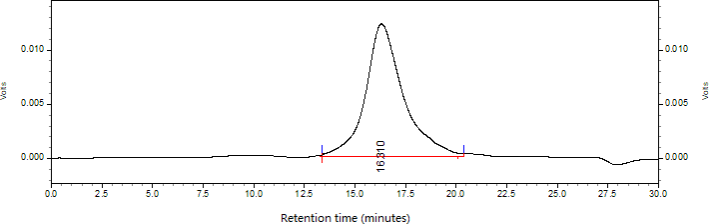
The profile of ALP on HPGPC

**FIGURE 2:**
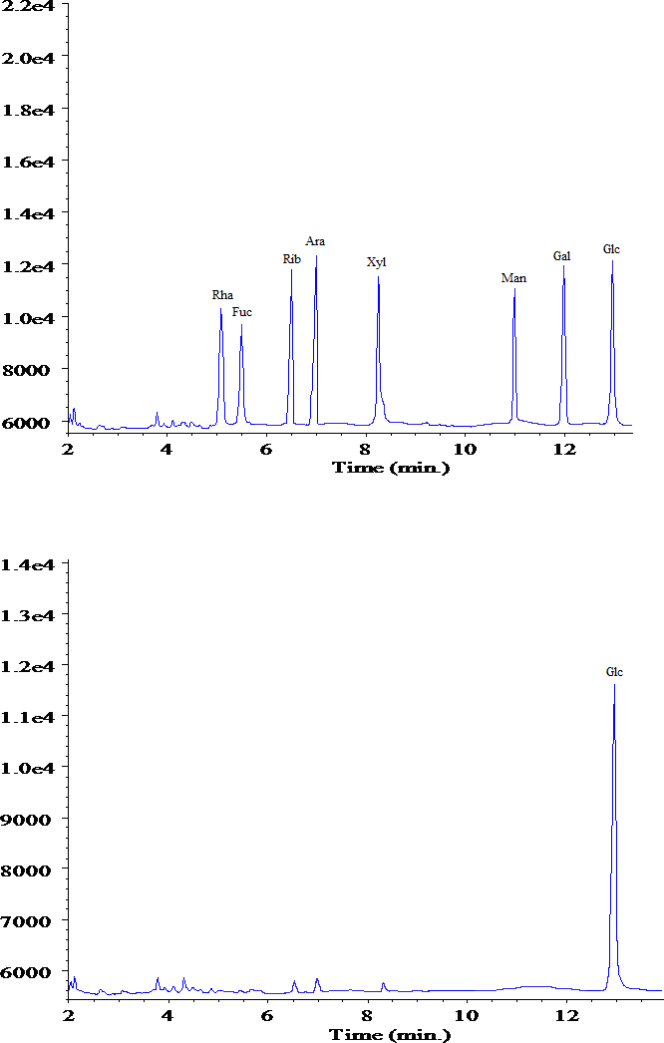
Gas chromatograms of the mixed standard monosaccharides and ALP. Ara, Arabinose; Fuc, Fucose; Gal, Galactose; Glc, Glucose; Man, Mannose; Rha, Rhamnose; Rib, Ribnose; Xyl, Xylose

The crude polysaccharides CALAP were extracted from the defatted rhizomes of *A lancea* by 2% NaOH extraction, hydrochloric acid neutralization, protein‐removing, ethanol precipitation and vacuum drying, with a yield of 5.23% of the dry raw material. The supernatant of CALAP was further stepwise isolated by anion‐exchange chromatography on a DEAE Sepharose Fast Flow column eluting with 0‐1 M NaCl aqueous solution at a flow rate of 2.0 mL/min and gel filtration chromatography on a Sephacryl S‐300 HR column eluting with 0.15 M NaCl at a flow rate of 1 mL/min, yield only one purified polysaccharide ALP from distilled water elution (yield of 0.12%). The ALP contained 96.23% carbohydrates and no uronic acid as determined by phenol‐sulphuric acid assay and the m‐hydroxydiphenyl method, indicating it is a neutral polysaccharide. A negative response to Bradford's method and no absorption at 280 nm in UV spectrum suggested ALP was free of proteins. The profile of ALP on HPGPC (Figure 1) showed a single, symmetrical, narrow peak, which indicated that ALP was a homogeneous polysaccharide. The average molecular weight of ALP was estimated to be 6.63 × 10^4^ Da as reference to the standard calibration curve made with T‐series Dextran (logMw = 10.67‐0.3586t, *R* = 0.9828, *t* = 16.310). GC analysis revealed that ALP was only composed of glucose, indicating that ALP was a glucan (Figure 2).

### 3.2 Expression of sLe^x^ antigen on the surface of U‐2 OS cells

**FIGURE 3:**
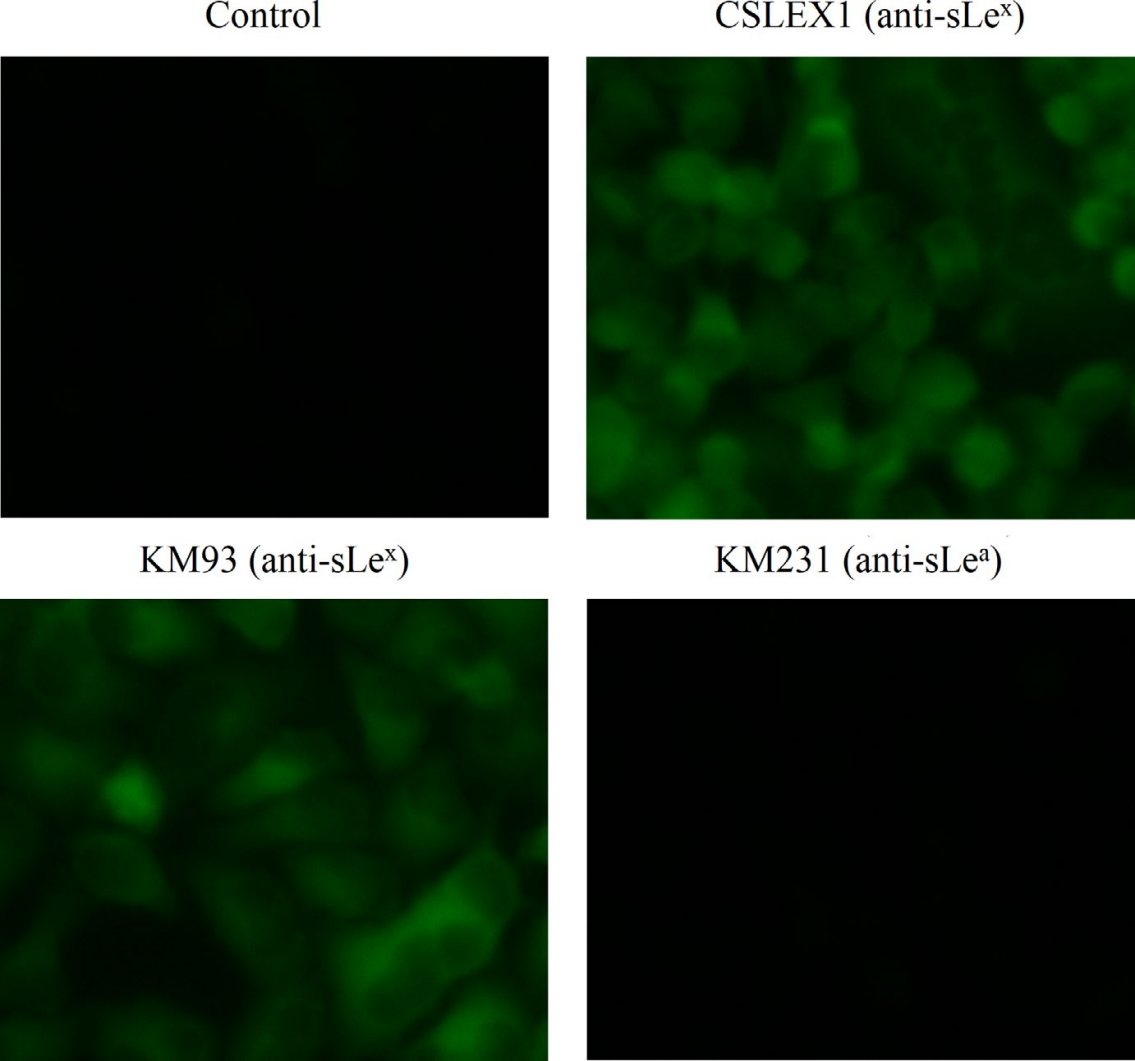
The expression pattern of sLe^x^/sLe^a^ antigens on U‐2 OS cells

As shown in Figure 3, only sLe^x^ was obviously seen on U‐2 OS cells as compared with the control sample without the addition of the antibody, while no sLe^a^ was expressed. This observing indicated that sLe^x^ antigen was present on the surface of U‐2 OS cells.

### 3.3 ALP from *A lancea* inhibits adhesion of U‐2 OS cells to HUVECs

**FIGURE 4:**
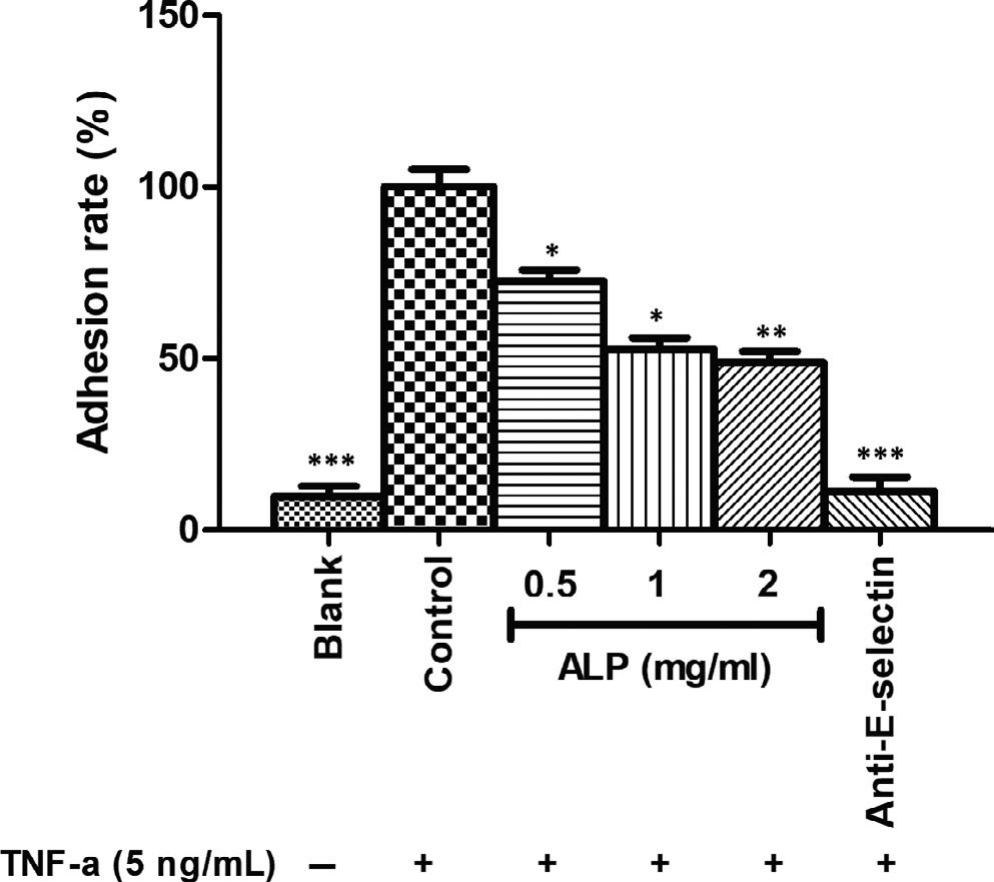
The effect of ALP from *Atractylodes lancea* on the TNF‐α‐induced adhesion of U‐2 OS to HUVECs. All data are represented as the means ± SD (n = 3). *P < 0.05, **P < 0.01 and ***P < 0.001 vs control

As shown in Figure 4, the maximum adhesion effect was observed in cell upon stimulating HUVECs with TNF‐a (5 ng/mL) and this binding was significantly reversed by the addition of anti‐E‐selectin antibodies, indicating that tumour adhesion mainly depends on ligand‐receptor interactions. Pretreatment with ALP (0.5, 1 and 2 mg/mL) showed different suppressing ability for adhesion of U‐2 OS cells to HUVECs with adhesion rate of 72.57% at 0.5 mg/mL, 52.86% at 1 mg/mL and 48.39% at 2 mg/mL, respectively. This result revealed that the inhibitory effect of ALP on cell adhesion between U‐2 OS cells and HUVECs.

### 3.4 ALP from *A lancea* suppresses cell invasion and migration of U‐2 OS cells in vitro

**FIGURE 5:**
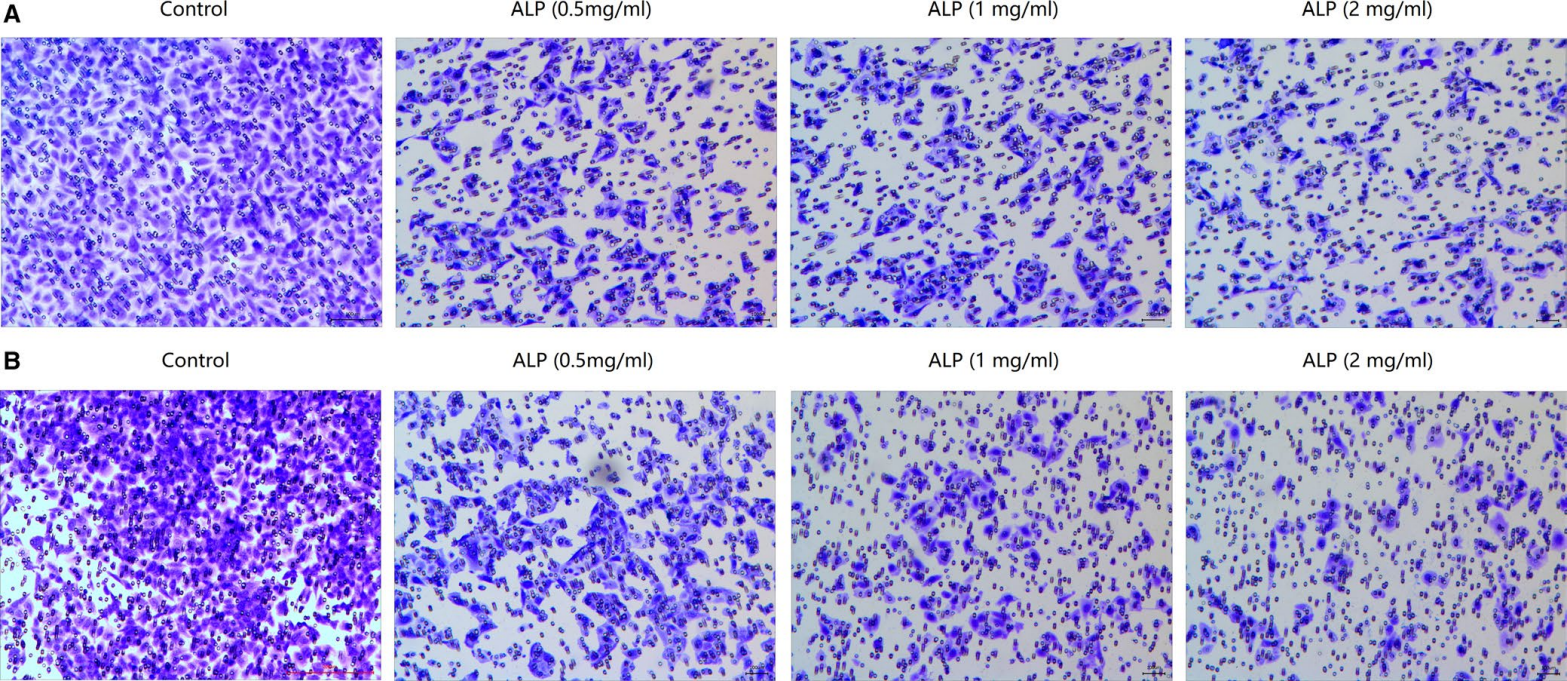
The effect of ALP from *Atractylodes lancea* on the metastasis of U‐2 OS cells. A, The effect of ALP from *A lancea* on the invasion of U‐2 OS cells. B, The effect of ALP from *A lancea* on the migration of U‐2 OS cells

As seen from Figure 5A, a significant loss of cells number invading to the lower surface of HUVECs membrane, as evidenced by the attenuation of crystal violet intensity, was observed in cells treated by ALP, which was present in a dose‐dependent manner. The same result occurred in the migration assay for ALP (Figure 5B). Both results suggested that ALP was able to effectively inhibit the invasion and migration of U‐2 OS cells in vitro.

### 3.5 ALP from *A lancea* inhibits sLe^x^ expression of U‐2 OS cells

**FIGURE 6:**
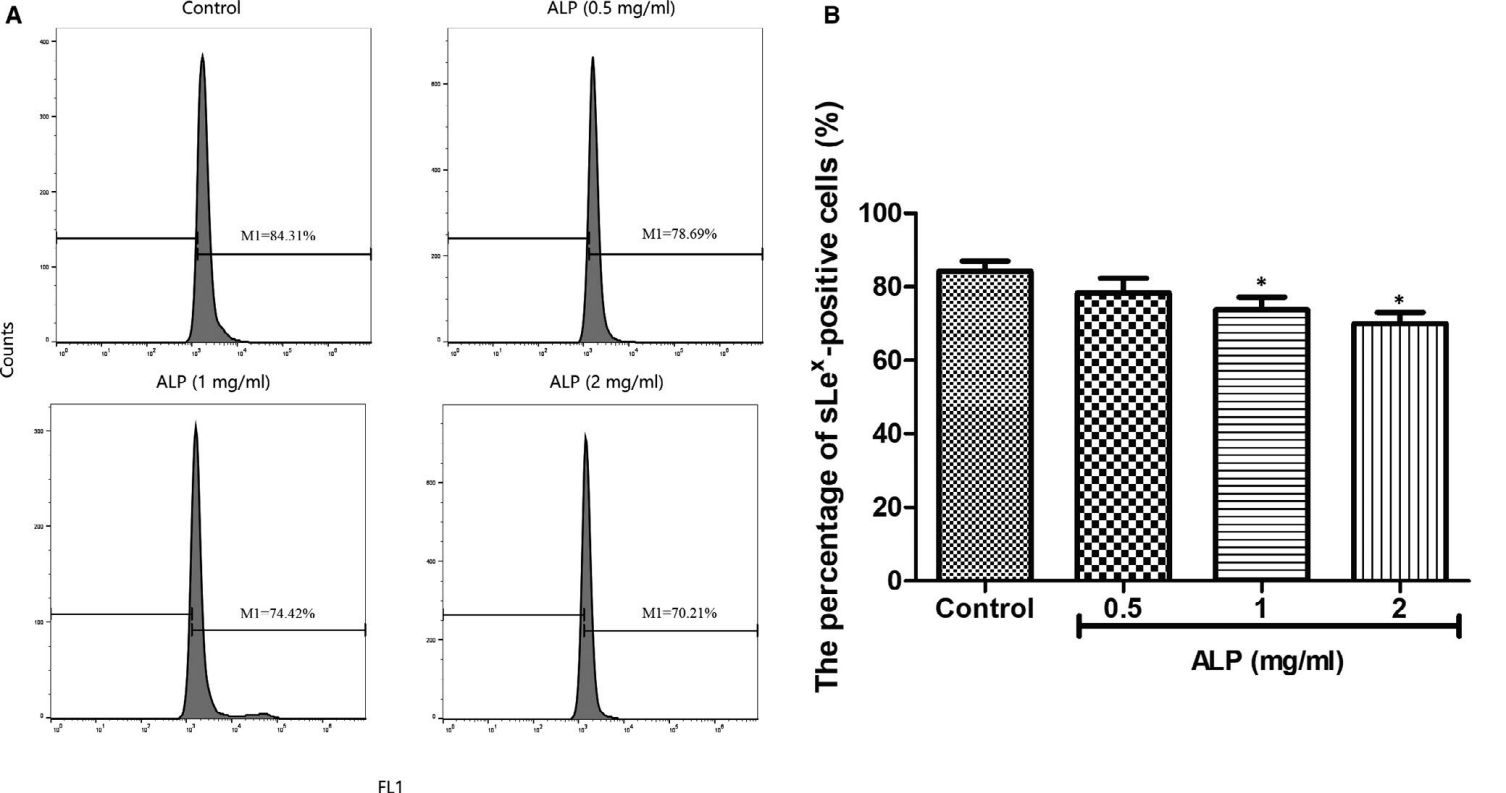
Flow cytometry analysis of sLe^x^ expression of U‐2 OS cells in response to ALP treatment or not. A, Flow cytometry analysis of sLe^x^ expression of U‐2 OS cells in response to ALP treatment or not; (B) the percentage of sLe^x^‐positive cells (%) in U‐2 OS cells in response to ALP treatment or not. All data are represented as the means ± SD (n = 3). ^*^ *P* < 0.05 and ^**^ *P* < 0.01 vs control

The result from flow cytometric analysis (Figure 6A,B) showed that untreated U‐2 OS cells had more sLe^x^ expression, whereas pretreatment with ALP (0.5, 1 and 2 mg/mL) resulted in a decreased number of the cells expressing sLe^x^ in a dose‐dependent manner, especially at the concentrations of 1 and 2 mg/mL when compared with control cells (*P* < 0.05).The present study verified that the cell adhesion inhibitory effect of ALP on U‐2 OS cells to HUVECs was achieved by mitigating the sLe^x^ expression on U‐2 OS cells.

### 3.6 ALP from *A lancea* induces apoptosis of U‐2 OS cells in vitro

**FIGURE 7:**
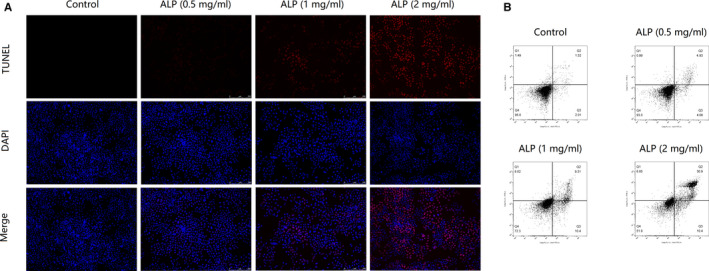
The effect of ALP from *Atractylodes lancea* on the apoptosis of U‐2 OS cells. A, Intracellular apoptotic nuclei was detected by TUNEL assay in U‐2 OS cells. B, Cell apoptosis was detected by Annexin V‐FITC/PI dual‐labelling technique by flow cytometry in U‐2 OS cells

TUNEL staining assay (Figure 7A) indicated that the addition of ALP led to a significant increase of apoptotic cells than that of the control (*P* < 0.01), as showed by the increased number of purplish red‐stained TUNEL‐positive nuclei. As illustrated in Figure 7B, the result of Annexin V‐FITC/PI double staining on flow cytometry further confirmed that ALP was found to induce apoptosis in U‐2 OS cells in a concentration‐dependent manner.

## 4 DISCUSSION

Cancer metastasis, as one of the major causes of cancer‐associated mortality, involves a multiple of cell movements, that is cancer cell adhesion, invasion, migration and circulation in blood and lymph. In this process, tumour cells detach from the primary tumour, penetrate secondary host organs and finally result in new tumour colonies.^25^, ^26^ Of these events, the attachment of cancer cells to human epithelial cells is an important stage in the initiation of metastasis, which leads to the development of neoplastic diseases, and it depends on specific cell‐surface adhesion molecule (CAM)‐mediated cell‐cell recognition.^27^, ^28^ Among CAM, E‐selectin appears to be one of critical molecules expressed on activated endothelial cells and are likely to bind its cell‐surface counter receptors sLe^x^ [NeuAcα2‐3Galβ1‐4(Fucα1‐3)GlcNAc‐R] on cancer cells.^29^ It is well noted that binding between E‐selectin and sLe^x^ plays a crucial role in tumour cell adherence to endothelial cells in the process of metastasis.^6^ To overcome this situation, molecules specifically designed to block their binding may have potential as antitumour agents.

A new water and alkaline‐soluble polysaccharide (ALP), with an average molecular weight of 6.63 × 10^4^ Da, was successfully isolated and purified from the defatted rhizomes of *A lancea*. GC analysis demonstrated that ALP was only composed of glucose, indicating it is a kind of glucan. With the aim of developing a potential antitumour alternative, the effect of ALP on E‐selectin/sLe^x^‐mediated adhesion between U‐2 OS cells to HUVECs was examined. With the help of using mAbs and FITC‐conjugated goat antimouse IgM/IgG secondary antibodies, the quantization of U‐2 OS cells expressing sLe^x^ oligosaccharides (namely fluorescent cells) was measured by fluorescence microscopy under an electron microscope. As anticipated, sLe^x^ was expressed on U‐2 OS cells. To investigate if the sLe^x^/E‐selectin mediated tumour adhesion to HUVECs was blocked by ALP in U‐2 OS cells, we examined the adhesion rate of U‐2 OS cells to HUVECs in response to different ALP treatment (0.5, 1 and 2 mg/mL) or not. It was apparent that ALP exhibited a potent inhibitory effect on the adhesion between U‐2 OS cells and HUVECs. In view of the above anti‐adhesive effect of ALP on U‐2 OS cells to HUVECs, we next examined the abilities of ALP (0.5, 1, and 2 mg/mL) on the cell invasion and migration of U‐2 OS cells using a modified transwell assay coated with a continuous HUVECs monolayer or not. In line with anti‐adhesive activity of ALP, the invasion and migration rate of U‐2 OS cells were significantly attenuated by the addition of ALP at dose of 0.5, 1 and 2 mg/mL. In this regard, flow cytometric analysis was conducted to detect the sLe^x^ expression in U‐2 OS cells upon different ALP treatment. The results indicated that the percentage of the cells expressing sLe^x^ was significantly decreased by ALP in a dose‐dependent manner, suggesting that the cell adhesion inhibitory effect of ALP on U‐2 OS cells to HUVECs was achieved by mitigating the sLe^x^ expression on U‐2 OS cells. Substantial experimental evidence indicated that anti‐metastasis effect of natural drug was usually accompanied by the induction of apoptosis in cancer cells.^30^, ^31^ Due to the close relationship between metastasis and apoptosis, we next explore if ALP induce apoptosis in U‐2 OS cells with TUNEL staining and Annexin V‐FITC/PI dual‐labelling technique by flow cytometry. Both assays demonstrated that ALP can induce apoptosis in U‐2 OS cells in a concentration‐dependent manner.

## 5 CONCLUSION

The present experimental results revealed that the adhesion, migration and invasion of U‐2 OS cells to HUVECs were significantly inhibited by ALP. It is possibly inferred that ALP is prone to bind E‐selectin instead of sLe^x^, thus hindering the combination of E‐selectin and sLe^x^ to promote tumour metastasis. In addition, U‐2 OS cells underwent the apoptosis following ALP treatment.

## CONFLICT OF INTEREST

The authors declare that they have no competing interests.

## AUTHOR CONTRIBUTION

**Kaihua Zhong:** Conceptualization (equal); data curation (equal); formal analysis (equal); funding acquisition (equal); supervision (equal); writing – original draft (equal). **Shuxin Fan:** Data curation (equal); methodology (equal); software (equal); validation (equal); visualization (equal); writing – original draft (equal). **Shujun Yao:** Data curation (equal); formal analysis (equal); investigation (equal); resources (equal); validation (equal). **Haibin Xu:** Conceptualization (equal); data curation (equal); formal analysis (equal); investigation (equal); methodology (equal); validation (equal); visualization (equal). **Suping Bai:** Conceptualization (equal); funding acquisition (equal); supervision (equal); writing – original draft (equal); writing – review and editing (equal).

## Data Availability

All data generated or analysed during this study are included in this article.
